# Letters to the Editor: Negative Pressure Dressing: Some Background to a Monopole Business

**Published:** 2008-02-15

**Authors:** A. M. Danino, E. Coeugniet

**Affiliations:** Professeur agrégé UM Service de chirurgie plastique du Centre Hospitalier de I'Université de Montréal, Hôpital Notre Dame, 1560 sherbrooke est. Montréal Quebec H2L4M1 Université de Montréal Québec Canada; Service de chirurgie plastique chru lille 59000, Lille cedex

I read with great of interest the article entitled “Application of Vacuum-assisted Therapy in Postoperative Ascitic Fluid Leaks” in the *Journal of Burns and Wounds*, April 2007, by Stawicki et al.

First of all, we congratulate the authors for their valuable work in the field of wound healing.

Also we want to draw attention to a few points about the negative pressure dressing so as to remain independent of the business world.

The negative pressure dressing is an external device that creates a vacuum on a wound.

This technique has 3 mechanisms of action:

- reduction of oedema and exudation with improvement of the local blood flow,
- reduction of pathogen bacterial colonization, and
- induction of tissue granulation by mechanical stress, causing increased rate of mitosis and neovascularization.

Over the past 10 years this technique has become more popular for wound care management because of a series of publications by Morykwas et al, the first article was published in *Annals of Plastic Surgery* 1997.^1^

Trends are common in medicine. Indeed major discoveries are rare and often ancient methods are just modified and published by a new author.

The special feature of the present negative pressure dressing trend is that it is driven by a business monopole.

The “refrigerator syndrome” is well known to all business and management students. The principle is to transform a nonspecific product or procedure into a brand name, which then becomes synonymous with the product or procedure. Examples of this include the 1960's commercialization of the refrigerator under the brand Frigidaire™ and more recently IBM and Microsoft for the “PC,” personal computer.

When this principle is applied to a medical device, our treatment, which should be selected in a free and unbiased manner, may be compromised.

One would like to think that the medical profession is better protected against commercialization than the rest of the population but, over a few years, the commercial name VAC (vacuum assisted closure) from the KCI company has taken the place of all nonspecific negative pressure dressings.

In the beginning, the advertising and promotion relied exclusively on scientific publications. These publications completely excluded the history of negative pressure dressings and defined the inception of the procedure with the production of the first VAC device. The medical community had been led to believe that this was a new scientific discovery.

As a result, we feel it necessary to provide an account of the negative pressure dressing in the history of wound care management.

The negative pressure dressing has been used since the 19th century for wound care purposes. Initially, a device to create negative pressure was used to allow difficult thoracic operations while avoiding collapse of the lungs.

Around 1905 a new technique was born. The patient's head protruded outside a negative pressure chamber while the patient's body, together with the surgeon, were inside the chamber.

The machine was then miniaturized by the surgeons during the World War I. Dr Sauerbruch, born in 1875 in Barnem, Germany, invented a portable bell which, put over the chest, isolated the thorax and the surgeons' hands only. Here is the description of its first use according to his memories^2^

the most difficult part was to supply this glass bell with an air absorbing pump (…). We prolonged our evening by calculating everything and in the daylights both boys went their ways. They first visited the glassworker, the second looked for rubber and valves. (…)

At the moment that I could slide my hands into the drum, manipulate freely my instruments inside, without air leak from outside, the preliminary experimental conditions seemed to be satisfied. Our first victim was Cesar, a very small hairy stray dog, who loved one of the lab boys. (…)

Several clinical notes from the same author in his autobiography describe further refinements to the bells allowing the treatment of infected wounds, especially on legs.

In more recent times, the technique was used by Russian surgeons during the 1970s.^3^,^4^ The principle was to apply a transparent flexible top under which a vacuum was created mostly by wall suction.

This bell mechanism was adapted by Blue Sky Medical in 2003 as the “Miller Dermi Vex”™ procedure.

In 1986, Kostiuchenok et al.^5^ demonstrated in a control study on 90 persons, the superiority of surgical debridement of infected wounds after negative pressure dressing compared with surgical debridement alone.

Davydov et al in 1986 demonstrated the use of the negative pressure dressing for purulent lactation mastitis on a series of 97 patients.^6^

In 1989, Chariker et Jeter published a negative pressure dressing method connecting wall suction via a silicon drain, on simple dressing gauze and a self-adherent semipermeable membrane covering the wound.

Again this procedure was bought by Blue Sky Medical in 2003 under the name of “Chariker-Jeter®technique.”^7^

The worldwide acceptance of the method is due work by Morykvas et al, who in 1987 developed a system, subsequently commercialized by KCI, including a polyurethane sponge and distributing negative pressure by intermittence or continuous suction through a machine.

Their approach involved repeating animal studies, especially on skin donor sites in pigs, and then producing a number of clinical publications based upon this research.

Since early 2000, the term negative pressure dressing was replaced in medical parlance by the trademark VAC. The former names topical negative pressure, sub-atmospheric pressure, vacuum sealing technique, and sealed surface wound suction have disappeared from the current vernacular. Worse still, all new scientific articles dealing with negative pressure dressings credit no research prior to the Morykwas et al publication of 1997.

To conclude, I would like to draw your attention to 2 things:

- Currently scientific publications generated by industry are being used like modern marketing to promote historically existing techniques as if they were novel.
- In this day and age, truly unique discoveries are unusual. Almost every “new” treatment is, in fact, a modification of a previously described technique. This is also true for wound treatments. The negative pressure dressing itself is a very useful and ancient method stemming from a historical continuum. For this reason it is important to understand the historical context, if we do not want to loose our critical mind.

Concerning wound healing, negative pressure dressing should be included with all other dressings: we should not mistake a particular commercial brand with a universal therapeutic procedure!
